# Biochemical and Structural Insights into FIH‐Catalysed Hydroxylation of Transient Receptor Potential Ankyrin Repeat Domains

**DOI:** 10.1002/cbic.202200576

**Published:** 2023-01-09

**Authors:** Benjamin G. Saward, Thomas M. Leissing, Ian J. Clifton, Anthony Tumber, Christopher M. Timperley, Richard J. Hopkinson, Christopher J. Schofield

**Affiliations:** ^1^ Department of Chemistry and the Ineos Oxford Institute for Antimicrobial Research Chemistry Research Laboratory Mansfield Road University of Oxford Oxford OX1 3TA UK; ^2^ CBR Division Defence Science and Technology Laboratory (DSTL) Porton Down Salisbury SP4 0JQ UK; ^3^ Present address: Leicester Institute for Structural and Chemical Biology and School of Chemistry University of Leicester Henry Wellcome Building, Lancaster Road Leicester LE1 7RH UK

**Keywords:** ankyrin, factor inhibiting HIF (FIH), hypoxia inducible factor HIF, oxygenases, demethylases, post translational modifications, transient receptor potential (TRP) channel

## Abstract

Transient receptor potential (TRP) channels have important roles in environmental sensing in animals. Human TRP subfamily A member 1 (TRPA1) is responsible for sensing allyl isothiocyanate (AITC) and other electrophilic sensory irritants. TRP subfamily vanilloid member 3 (TRPV3) is involved in skin maintenance. TRPV3 is a reported substrate of the 2‐oxoglutarate oxygenase factor inhibiting hypoxia‐inducible factor (FIH). We report biochemical and structural studies concerning asparaginyl hydroxylation of the ankyrin repeat domains (ARDs) of TRPA1 and TRPV3 catalysed by FIH. The results with ARD peptides support a previous report on FIH‐catalysed TRPV3 hydroxylation and show that, of the 12 potential TRPA1 sequences investigated, one sequence (TRPA1 residues 322–348) undergoes hydroxylation at Asn336. Structural studies reveal that the TRPA1 and TRPV3 ARDs bind to FIH with a similar overall geometry to most other reported FIH substrates. However, the binding mode of TRPV3 to FIH is distinct from that of other substrates.

## Graphical abstract

**Confirmation analysis**: Following the differing conclusions of previous studies, we report biochemical and structural studies on asparaginyl hydroxylation of the ankyrin repeat domains of human transient receptor potential channels TRPA1 and TRPV3 as catalysed by the 2‐oxoglutarate oxygenase factor inhibiting hypoxia‐inducible factor (FIH).

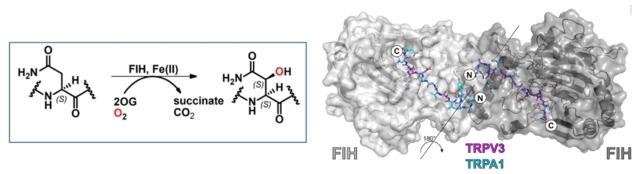

## Introduction

Transient receptor potential (TRP) proteins are calcium and sodium ion channels that enable cells to sense their external environments.^[1]^ TRP structures comprise 6 transmembrane (TM) helices, a pore forming region located between TM helices 5 and 6, and N‐ and C‐terminal cytoplasmic domains.[^2^, ^3^] Exposure to electrophiles such as allyl isothiocyanate (AITC) activates TRP subfamily A member 1 (TRPA1)^[4]^ through modification of a cysteine residue in its N‐terminal cytosolic domain.^[5]^ Activation of TRPA1 typically produces sensations of pain and irritation in mammals,[^6^, ^7^, ^8^] while a gain of function mutation in the *TRPA1* gene is responsible for familial episodic pain syndrome.^[9]^ Like TRPA1, TRP subfamily vanilloid member 3 (TRPV3) is activated by external stimuli including raised temperature and compounds such as carvacrol.^[10]^ Both TRPA1 and TRPV3 are expressed in the basal skin layers and TRPV3 also has a role in skin health.[^11^, ^12^, ^13^]

Factor inhibiting hypoxia inducible factor (FIH) is a dimeric Fe^II^‐ and 2‐oxoglutarate (2‐OG)‐dependent protein hydroxylase that plays a role in the regulation of the hypoxic response by catalysing hydroxylation of a conserved asparagine residue in hypoxia inducible factor α (HIF‐α) subunits.[^14^, ^15^, ^16^] This post‐translational modification reduces the interaction of transcriptionally active HIF with coactivator histone acetyl transferases (CBP/p300), potentially regulating HIF transcription in a gene‐ and context‐dependent manner.^[17]^

Evidence has also been presented that multiple ankyrin repeat domains (ARDs), interact with FIH, with many acting as substrates. Notably, residues other than asparagines (e. g., Asp and His) can be FIH substrates within ARDs,[^15^, ^16^, ^18^, ^19^] while, in some cases, FIH can catalyse two hydroxylations of the same ARD.^[20]^ Whereas the region of HIF‐α undergoing FIH‐catalysed hydroxylation is largely disordered in solution, ARDs adopt a well characterised canonical fold which is proposed to unwind to enable catalytically productive binding at the FIH active site.^[21]^ This proposal is supported by biophysical analyses including crystallographic analyses of FIH in complex with both HIF‐α and ARD fragments.[^15^, ^16^, ^18^, ^19^, ^22^, ^23^]

Evidence that TRPA1 might act as an oxygen/hypoxia sensor has been reported,[^21^, ^24^] which is of interest in part because of the role of TRP channels in temperature sensing (oxygen solubility decreases with temperature). TRPA1 is reported to be a substrate for the HIF‐α prolyl hydroxylases (PHDs),[^21^, ^24^] catalysis by which regulates HIF‐α levels in an oxygen‐dependent manner.^[25]^ However, studies with isolated PHDs have not supported this assignment.^[26]^

Karttunen et al. have also shown that FIH can catalyse hydroxylation of TRPV3 in studies on isolated components and in cells, although in the latter case, hydroxylation has not yet been demonstrated with endogenous TRPV3.^[19]^ The residue in TRPV3 proposed to undergo FIH‐catalysed hydroxylation is Asn‐242, which is in the TRPV3 cytoplasmic ARD. Importantly, Karttunen et al. provided evidence that FIH‐catalysed hydroxylation of TRPV3 inhibits TRPV3 activity.^[19]^ Here we report biochemical and structural insights into the FIH‐catalysed hydroxylation of TRPA1 and TRPV3, the results of which support the work of Karttunen et al. on TRPV3 and indicate the potential for hydroxylation of other TRP channel ARDs, including on at least one ARD of TRPA1.

## Results

### TRPA1‐derived fragment (322–348) is efficiently hydroxylated by FIH

**Figure 1:**
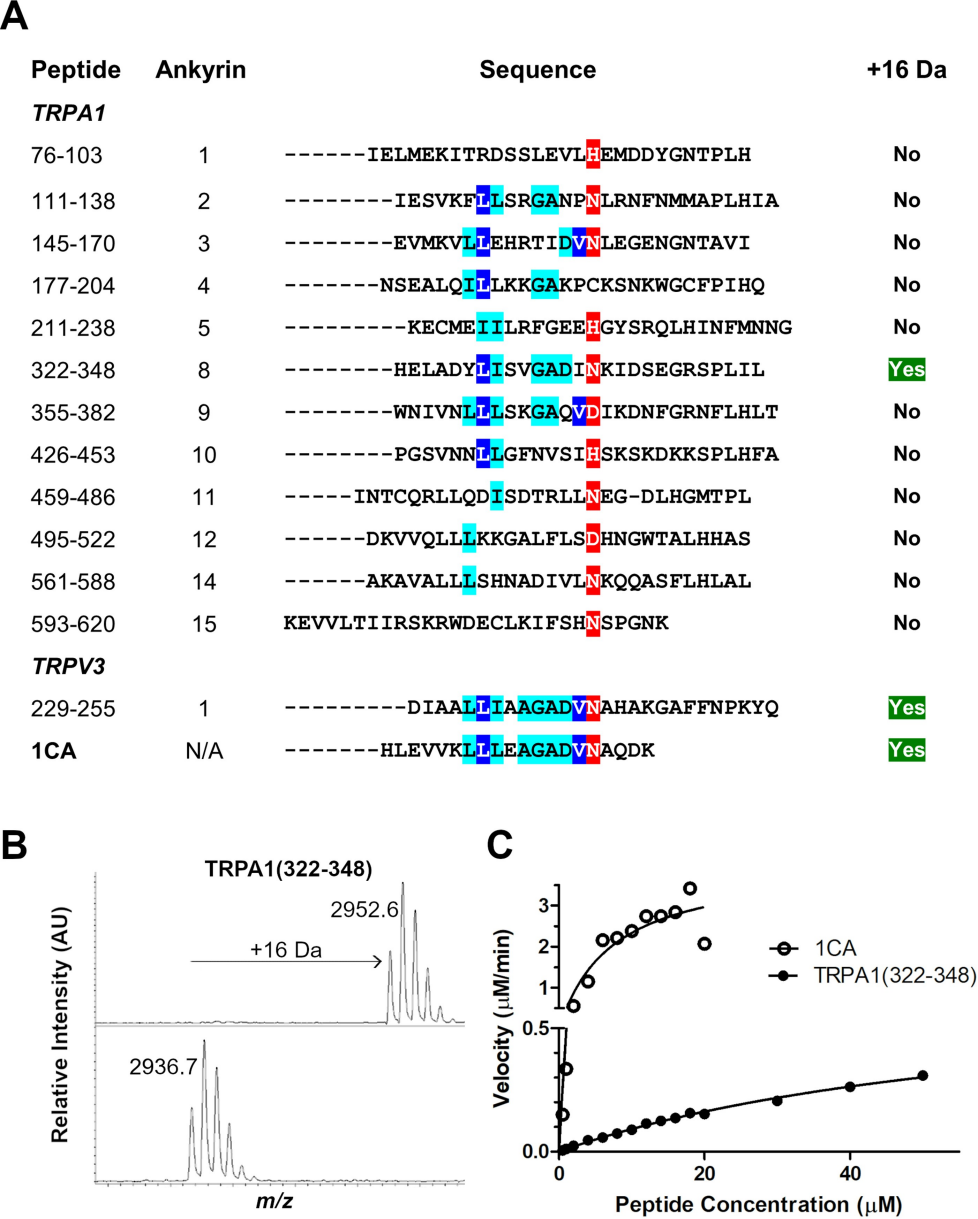
FIH catalyses the hydroxylation of ankyrin repeat domain fragments from TRPA1 and TRPV3. A) Alignment of TRPA1‐ and TRPV3 (residues 229–255)‐derived peptides with the 1CA consensus ARD sequence.^[28]^ B) Matrix‐assisted laser desorption (MALDI) MS spectra showing a +16 Da mass shift of 10 μM TRPA1 (322–348), as catalysed by FIH. Conditions: FIH (2 μM, top panel; 0 μM, bottom panel), sodium ascorbate (100 μM), 2‐OG (100 μM), ferrous ammonium sulfate (20 μM) in 50 mM Tris buffer (pH 7.5, 37 °C, 1 h). C) Comparison of hydroxylation of peptides by FIH as assessed by SPE‐MS. Conditions: FIH (0.1 μM, 1CA; 0.4 μM, TRPA1 (322–348)), sodium ascorbate (100 μM), 2‐OG (100 μM), ferrous ammonium sulfate (20 μM) in 50 mM Tris buffer (pH 7.5, RT). Error bars represent SEM for *n*=2 experiments performed in triplicate.

To explore the scope of FIH‐catalysed TRPA1 channel ARDs, we synthesised a panel of 12 putative ankyrin loop‐containing peptides derived from TRPA1 for use in FIH‐mediated hydroxylation assays (Figure 1A).^[27]^ The reported consensus ankyrin sequence (1CA) was used as a positive control (Figure S1 in the Supporting Information).^[28]^ The previously reported TRPV3 substrate was also tested under our assay conditions and was found to undergo a single hydroxylation (Figure 1A).^[19]^ This observation contrasts with the lack of activity observed for the putative TRPA1 HIF prolyl hydroxylase substrate.^[26]^ Whilst there was no clear evidence for hydroxylation of 11 of the 12 tested TRPA1 ARD peptides, it was found that one TRPA1‐derived peptide (322–348) was hydroxylated once by FIH (2 μM enzyme at 37 °C, Figures 1B and S1), albeit with less efficiency than 1CA (specific activity 0.026 μM^−1^ min^−1^ compared to 5.50 μM^−1^ min^−1^ for 1CA, Figures 1C and S1). Analysis of the hydroxylated TRPA1‐derived peptide (322–348) showed that hydroxylation occurs at a single asparagine residue (Asn336, Figure S2).

### Crystallographic analysis of TRPA1‐derived peptide with FIH

**Figure 2:**
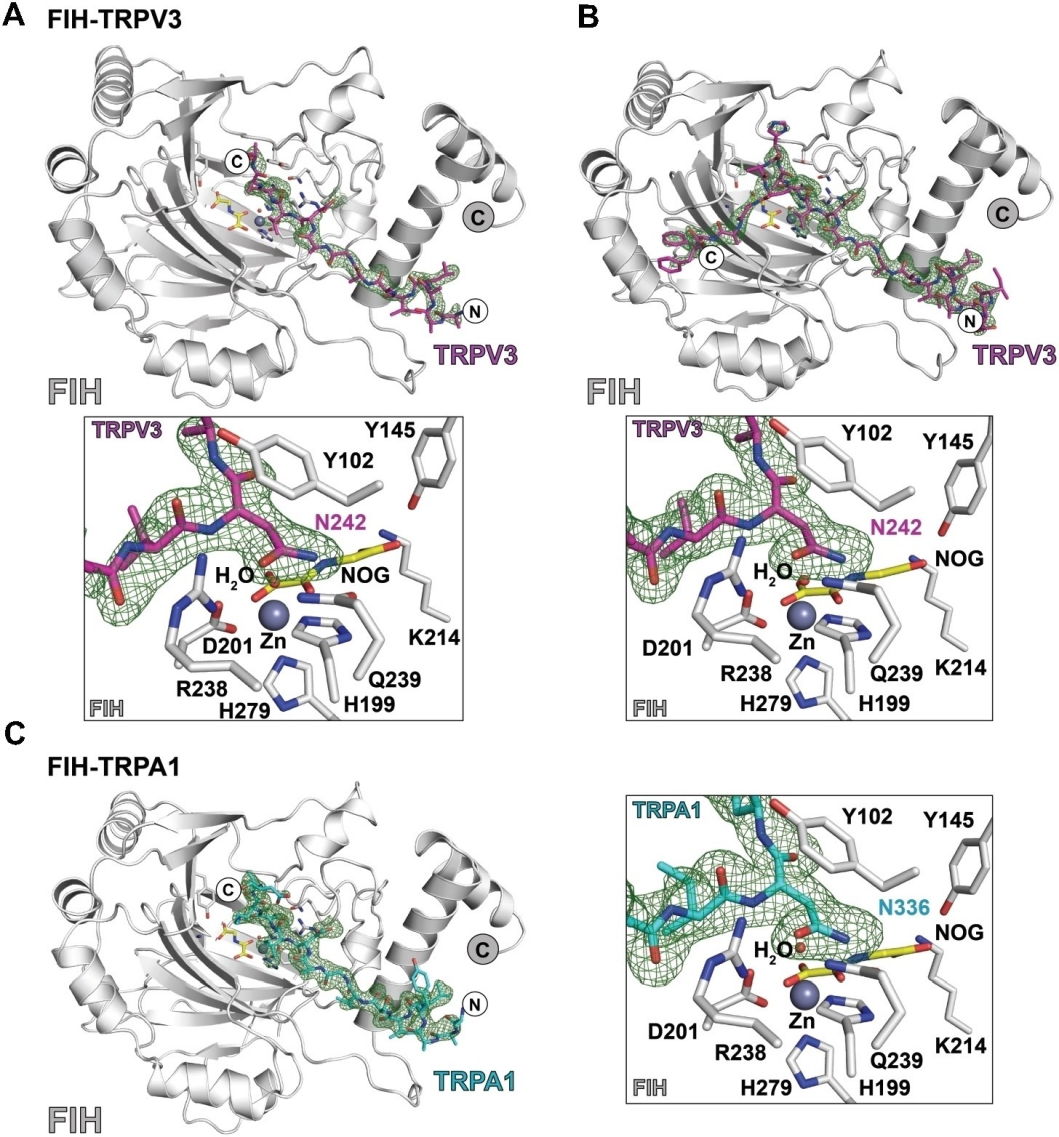
Views with electron density maps of crystal structures of FIH in complex with TRPA1‐ and TRPV3‐derived peptides. Fo−Fc OMIT maps are contoured to 3*σ* and are carved around the substrates. A) FIH‐Zn/TRPV3 Ank1 (220–246) (PDB ID: 6HA6); B) FIH‐Zn/TRPV3 Ank1 (229–255) (PDB ID: 6H9J); C) FIH‐Zn^II^/TRPA1 Ank8 (313–339) (PDB ID: 6HC8).

**Figure 3:**
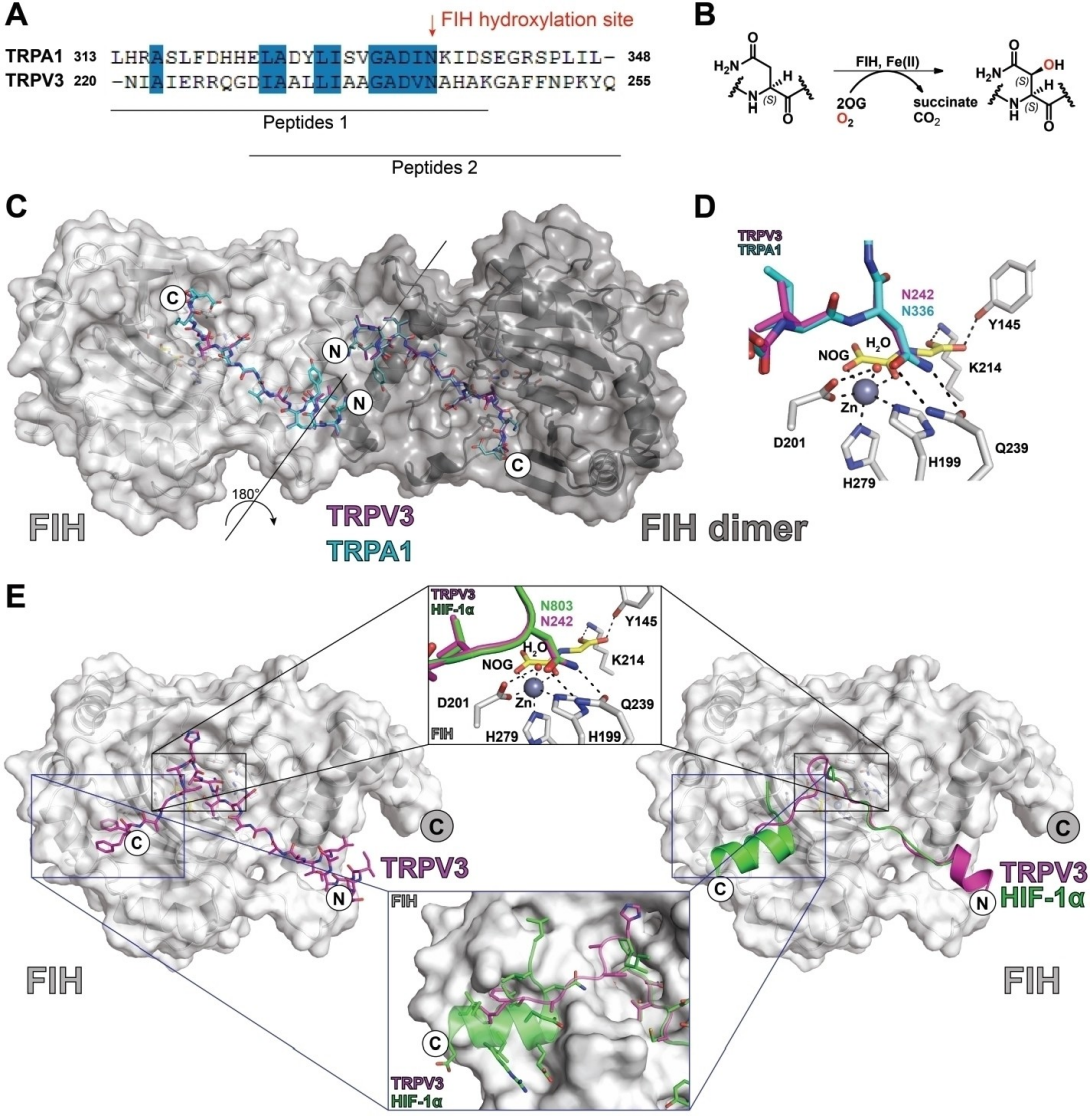
Views of crystal structures of FIH in complex with TRP channel‐derived peptides. A) Sequence alignment of TRP channel FIH substrates used for crystallography. B) Scheme for FIH‐mediated Asn‐residue hydroxylation reactions. C) Overlay of crystal structure views of FIH in complex with TRPV3 (220–246) and TRPA1 (313–339) peptides. The FIH dimer is in dark grey. D) Overlaying views from structures of FIH complexed with TRPA1 (220–246) and TRPV3 (220–246) peptides reveals near‐identical binding modes at the active site. E) Comparison of a structure of FIH complexed with TRPV3 (229–255) with a crystal structure of FIH in complex with HIF‐1α (PDB ID: 1H2K)^[29]^ indicating differences in their crystallographically observed binding modes.

To investigate whether TRP channel ARDs can bind to FIH in a similar fashion to HIF‐1α and other ARD proteins,^[29]^ we attempted to crystallise FIH in the presence of zinc(II), N‐oxalylglycine (NOG), and TRPV3 (220–246)‐ or TRPA1 (313–339)‐derived peptides. NOG is a near isostere of 2‐OG and is a broad spectrum 2‐OG oxygenase inhibitor (Figure 3D).^[30]^ Although successful in other cases, attempted co‐crystallisation of FIH with these two TRP channel ARD peptides was unsuccessful. However, after soaking the peptides into preformed FIH⋅zinc⋅NOG crystals, electron density corresponding to the peptides in the FIH active site was observed in both monomers in the FIH dimer (Figures 2 and 3A–E, Tables S1 and S2). The structures were solved by molecular replacement (using PDB ID: 2H2K).

Analysis of the resultant structures implies that both the TRPV3‐ and TRPA1‐derived substrates bind to FIH in a similar manner to that observed for the C‐terminal transcriptional activation domain of HIF‐1α, as also observed by crystallography (Figure 3E).^[29]^ In each case, the substrate asparagine residue is bound to FIH by interactions with Gln239 and Tyr102, while the isopropyl group of the −1 (relative to the hydroxylated asparagine) valine residue is positioned to interact in a hydrophic manner with the indole ring of Trp296 (Figures 2 and 3D, Figure S3). The sidechain of the −8 leucine residue binds in a hydrophobic pocket at the FIH dimerization interface, as observed with HIF‐1α (Figure 3C and E).[^29^, ^31^] However, only the fourteen C‐terminal residues of TRPV3 (220–246) peptide and the 16 C‐terminal residues of the TRPA1 (313–339) peptide were visible in the respective crystal structures, suggesting that the N‐terminal residues of both substrate peptides are disordered in the crystalline state (Figure 3D and E). The visible N‐terminal region residues of the two substrate fragments in the dimer project towards each other at the FIH dimer interface region (Figure 3).

Biochemical studies with the HIF‐1/2α C‐terminal transcriptional activation domains and Notch1 substrates indicate that longer FIH substrates, especially those with additional residues to the C‐terminal side of the hydroxylation site that interact with FIH at the structurally defined substrate binding site two, can be more efficiently hydroxylated.[^29^, ^32^] To date, site two substrate binding to FIH has only been observed in crystal structures of FIH in complex with HIF‐1α peptides.^[29]^ To investigate the potential site two binding of TRPV3, FIH was co‐crystallised in the presence of Zn^II^, NOG, and a TRPV3 (229–255) peptide extended at its C terminus (Figure S3). The resultant crystal structure for the FIH⋅zinc(II)⋅NOG⋅TRPV3 (229–255) complex revealed clear density for the substrate in both the active site site one and site two substrate binding sites (Figure 2).

Comparison of crystal structures of FIH in complex with TRPV3 (220–246) and TRPV3 (229–255) show near‐identical and apparently productive substrate binding geometries in the Fe‐chelating active site region (Figure S3). Flexibility in the substrate binding mode was only observed at the termini of the peptide substrates, indicating the active site binding mode of the substrates in the crystal state is not substantially influenced by the substrate length or specific ARD sequence (at least as observed by crystallography).

## Conclusion

TRPA1 channel activity is reported to be regulated by catalysis by the HIF‐α prolyl hydroxylases (PHDs or EGLNs).^[24]^ However, prolyl hydroxylation of TRPA1 in cells requires further validation, as a subsequent study has shown a lack of activity of recombinant PHD1‐3 on reported non‐HIF substrates, including TRPA1.^[26]^ By contrast, our combined biochemical and structural studies support the report of FIH‐mediated ARD hydroxylation of TRPV3, and imply that FIH might catalyse hydroxylation of ARDs in other TRP channels, including TRPA1.^[19]^

FIH catalyses the hydroxylation of multiple residues (including asparagine, histidine and aspartate) on a range of ARD (and other) proteins.[^15^, ^16^, ^18^, ^19^, ^29^, ^31^] In addition to its ability to accept multiple substrates, FIH also catalyses different types of oxidative reactions including desaturations and protein crosslinking.[^33^, ^34^] It is thus notable that we only accrued clear evidence for hydroxylation of one of the 12 TRPA1 sequences we tested. Therefore, it appears that there may be a high degree of selectivity in FIH‐catalysed TRP ARD hydroxylation; it should, however, be noted that we only explored consensus type sequnces from TRPA1 as FIH substrates, and it is possible that FIH interacts with, and hydroxylates, other regions of TRPA1.

The physiologically relevant biological role, if any, of FIH‐catalysed TRP ARD hydroxylation is presently unknown. FIH‐catalysed hydroxylation of asparagine residues in ARDs can stabilise the ARD fold in isolated proteins,^[28]^ hence TRP channel hydroxylation might modulate the ability of regulatory proteins, for example, calmodulin,^[35]^ to bind to the ARD‐containing TRPA1N‐terminal cytosolic domain. However, studies on FIH KO mice have not revealed any clear link to HIF or TRP channel function, instead a phenotype with an increased metabolic rate and reduced mass manifested.^[36]^ There is also evidence that the roles of FIH in the HIF‐mediated hypoxic response are context‐dependent.[^37^, ^38^] The roles of FIH in the hypoxic response and TRP channel function, for example, in skin, thus might not have been manifest under the tested stresses placed on the FIH KO mice. However, given the lack of clear assignment of function for FIH‐catalysed hydroxylations of other ARD proteins, this possibility may be considered speculative.

Crystallographic studies on FIH in complex with TRPV3 and TRPA1 ARD fragments show binding in a manner related to that of HIF‐1α and previously studied ARD FIH substrates, though they suggest a binding mode of TRPV3 to FIH that might be distinct from that of other substrates away from the immediate active site region (Figures 2, 3 and S3). An extended TRPV3 fragment was observed to bind at substrate binding site 2 of FIH and in a manner somewhat different from that observed for the HIF‐1α C‐terminal transcriptional activation domain.^[29]^ It is possible that binding at site 2 helps to regulate the rates at which different substrates are hydroxylated by FIH. To date, no structure of ankyrin‐8 of TRPA1 has been reported; however, structural analysis of TRPV3 suggests that the ARDs of TRP channels (and other ARD FIH substrates) need, at least partially, to unfold in order to bind efficiently to FIH, as is the case for catalytically productive binding of other ARD proteins binding to FIH.[^17^, ^39^]

TRPA1 and TRPV3 assemble in homo‐ or hetero‐tetramers to form functional channels,[^27^, ^40^] an arrangement that potentially brings the ARDs of several TRPA1 and TRPV3 subunits into proximity. FIH forms a homodimer in solution that can, at least in the crystal state, bind two substrates simultaneously.[^29^, ^41^] Although no direct interactions between the two TRPV3/TRPA1 substrates simultaneously complexed with the FIH dimer were observed in our crystal structures (Figures 2 and S3), the N‐terminal regions of the two bound substrates come into close proximity (>7 Å). Thus, there is potential for the FIH dimer to simultaneously interact with two ARDs either from the same or different TRP channels.

Given the apparent complexity of the biochemistry and multiple substrate roles of FIH, which is highly conserved in higher animals,^[17]^ we suggest that the therapeutic potential of modulating FIH activity,^[42]^ including with respect to TRP channel activity, might be best explored by the development of potent small‐molecule inhibitors linked to disease‐relevant in vivo assays.

## Experimental Section

**Peptide synthesis and purification**: Peptides derived from TRPA1, TRPV3 and the 1CA consensus sequence^[28]^ were synthesised with C‐terminal amides using a CEM Liberty Blue solid‐phase peptide synthesis (SPPS) machine using Oxyma®/DIC‐mediated, microwave‐assisted couplings as reported.^[43]^ In brief, SPPS was performed using rink amide MBHA resin and 0.2 M solutions of FMOC‐protected amino acids in DMF. Deprotection of the N‐terminal FMOC group was performed using 20 % (*v/v*) piperizine in DMF and subsequent global deprotection was performed using a mixture of CF_3_CO_2_H, trimethylsilane, dimethoxybenzene and water. Purifications were carried out using a JASCO HPLC system fitted with a Phenomenix Gemini‐NX5C18 (30×250) column or on a Dionex Ultimate 3000 HPLC system fitted with a GRACE Vydac 218TP C_18_ (22×250 mm) column.

**Mass spectrometry**: MALDI‐MS and MS/MS spectra were obtained using an AutoFlex Speed machine equipped with a 96‐spot ground steel target (Bruker). Samples were mixed in a 1 : 1 (*v*/*v*) ratio with 10 mg/mL α‐cyano‐4‐hydroxycinnamic acid dissolved in 50 % (*v*/*v*) aqueous acetonitrile with 0.1 % (*v*/*v*) CF_3_CO_2_H. The target was allowed to dry at room temperature for 1 hour before being loaded into the spectrometer. MS/MS data were processed using Biotools (Bruker). Specific activities were determined using a RapidFire365 high‐throughput platform (Agilent) coupled to a 6550 quadrupole‐time of flight mass spectrometer (Agilent). Data were processed with Masshunter (Agilent) and Prism 5 (GraphPad Software Inc.).^[20]^

**Recombinant protein production**: Recombinant FIH was produced to high purity (<95 % by SDS‐PAGE analysis) from *Escherichia coli* BL21(DE3) cells as previously described.^[44]^ In brief, competent cells were transformed with a pET28a(+) plasmid encoding for the FIH gene and were grown in 2‐YT media supplemented with kanamycin. Expression was induced with isopropyl‐β‐d‐thiogalactoside at 37 °C for 4 h. FIH was purified by nickel affinity and size exclusion chromatography as reported.^[44]^

**X‐ray crystallography**: The peptide complex structures were produced by soaking the TRPV3 (220–246) or TRPA1 (313–339) peptides into preformed FIH⋅zinc(II)⋅NOG crystals. The FIH⋅zinc(II)⋅NOG⋅TRPV3 (229–255) complex structure was obtained by co‐crystallisation. Crystals were grown, unless otherwise stated, using the vapor diffusion technique at 20 and 4 °C in Intelli‐Plate 96–3 low‐profile plates (Art Robbins Instruments). Crystallisation plates were set up using a Rigaku Phoenix RE Drop setter instrument (Art Robbins Instruments). For aerobic crystallisation purposes, Fe^II^ was substituted with Zn^II^ to avoid metal oxidation. Crystals of the FIH⋅zinc(II)⋅NOG⋅TRPV3 (229–255) complex were cryo‐protected by transfer into the crystallisation buffer supplemented with 20 % (*v*/*v*) glycerol and freeze‐cooled by plunging into liquid nitrogen. Crystals of the FIH⋅zinc(II)⋅NOG complex were transferred into the crystallisation buffer supplemented with 20 % (*v*/*v* glycerol and either TRPV3 (220–246) peptide (2 mM final concentration) or TRPA1 (313–339) peptide (2 mM final concentration) for 14 h prior to freeze‐cooling by plunging into liquid nitrogen. X‐ray diffraction data were collected at Diamond Light Source (Didcot, UK; Table S1). Phases were solved by molecular replacement using Phaser/CCP4 (FIH PDB ID: 1H2K).^[29]^ Data were processed using *xia2* ^[45]^ and refined using PHENIX.^[46]^ Altering cycles of refinements using PHENIX and model building using COOT were performed until *R*_work_ and *R*_free_ converged. For more detailed statistical information, see Tables S1 and S2.

## Conflict of interest

The authors declare no conflict of interest.

1

## Supporting information

As a service to our authors and readers, this journal provides supporting information supplied by the authors. Such materials are peer reviewed and may be re‐organized for online delivery, but are not copy‐edited or typeset. Technical support issues arising from supporting information (other than missing files) should be addressed to the authors.

Supporting Information

## Data Availability

The data that support the findings of this study are available from the corresponding author upon reasonable request.
