# Supplementary material for: Biochemical and Structural Insights into FIH‐Catalysed Hydroxylation of Transient Receptor Potential Ankyrin Repeat Domains
Source: Chembiochem. 2023 Jan 9;24(4):e202200576. doi: 10.1002/cbic.202200576 (PMC10946520; doi:10.1002/cbic.202200576)
Supplement: Supplementary file 1 — Supporting Information [file CBIC-24-0-s001.pdf]

ChemBioChem

Supporting Information

Biochemical and Structural Insights into FIH-Catalysed Hydroxylation of Transient Receptor Potential Ankyrin Repeat Domains

Benjamin G. Saward[+], Thomas M. Leissing[+], Ian J. Clifton, Anthony Tumber, Christopher M. Timperley, Richard J. Hopkinson,* and Christopher J. Schofield*

Supplemental Information

Supplementary Figures

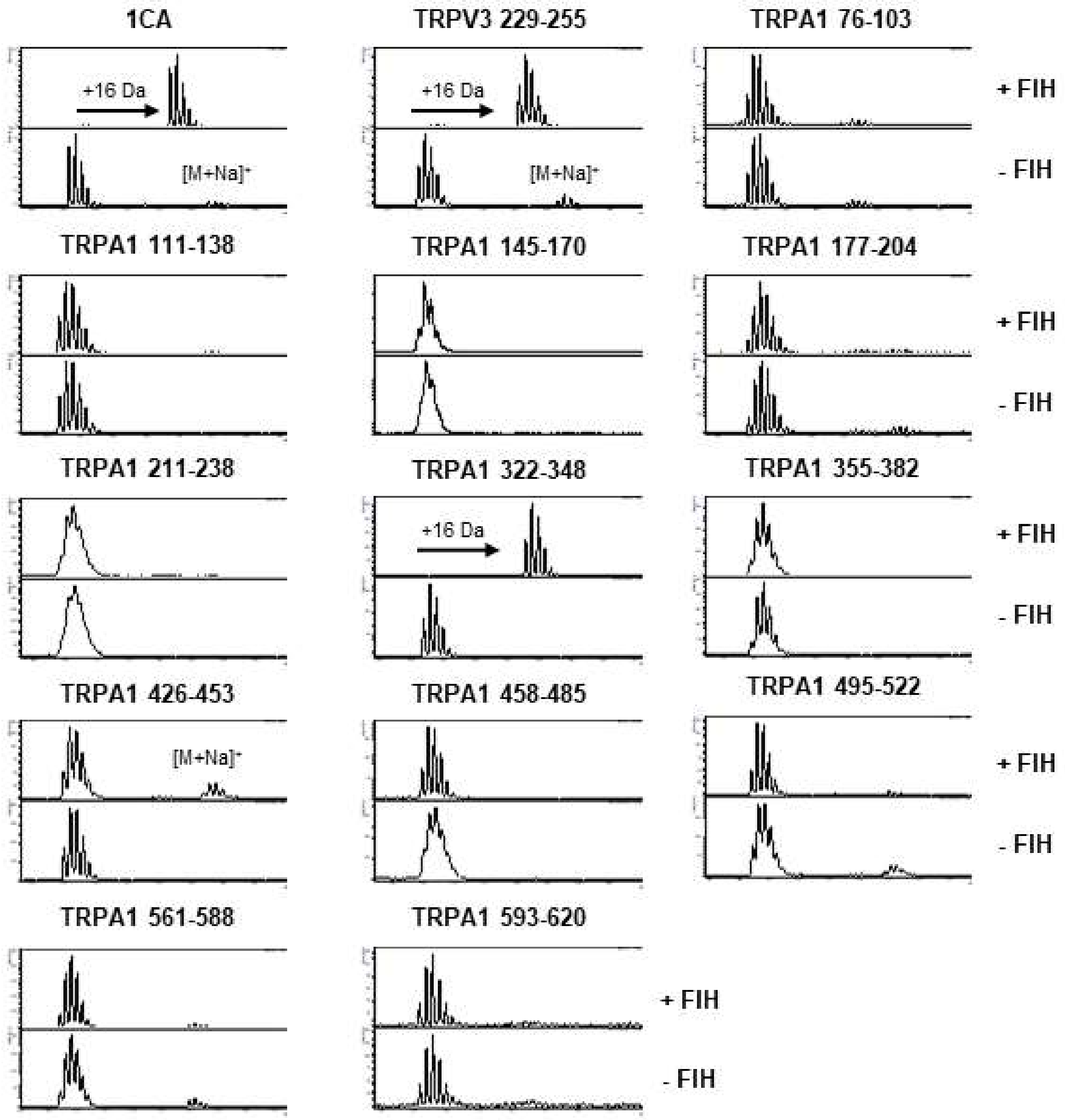

Figure S1: MALDI-MS spectra of peptides derived from TRPA1 and TRPV3 reacted with FIH. Assay conditions: 10 µM peptide, 20 µM ferric ammonium sulfate, 100 µM 2-oxoglutarate, 100 µM sodium ascorbate, 0 or 10 µM FIH, 50 mM Tris pH 7.5, 37 °C, 2 h. NB. TRPA1 ankyrin 8 (322-348) is the only TRPA1 derived peptide in this screen that undergoes hydroxylation by FIH. Peptide sequences are defined in Figure 1A.

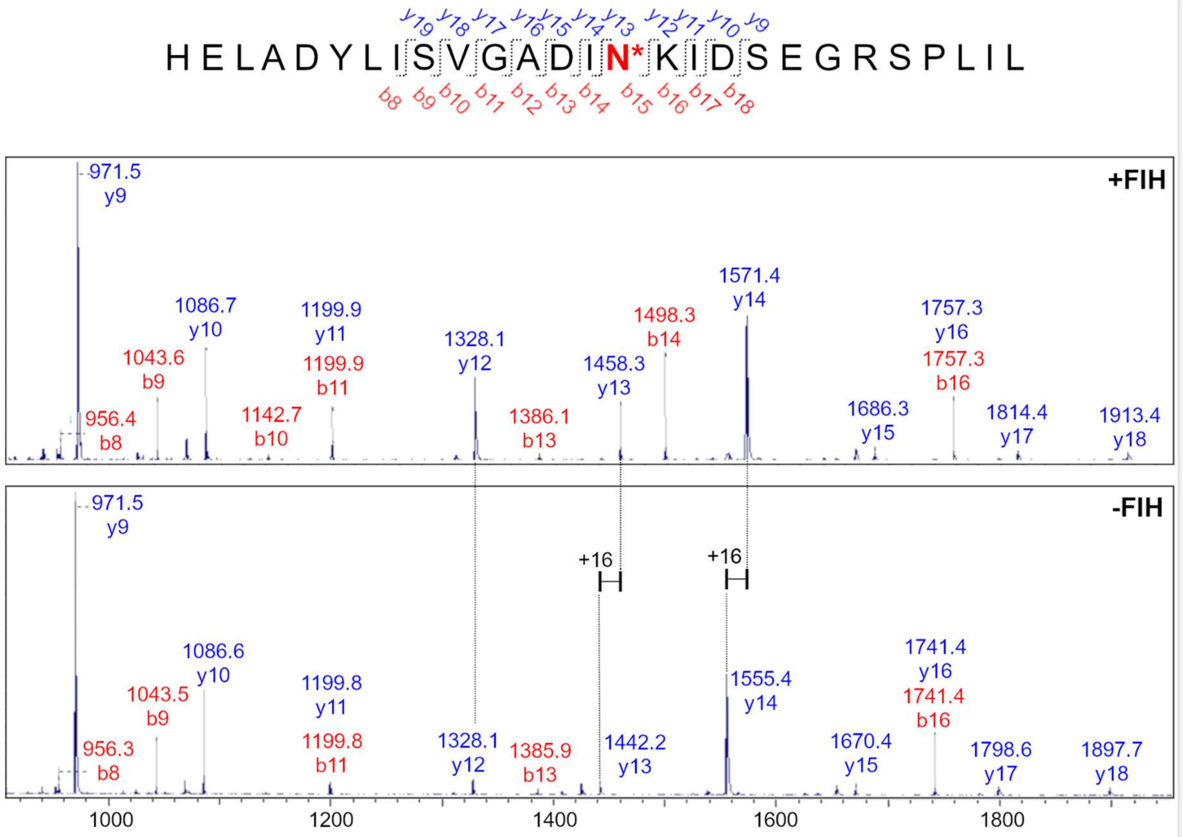

Figure S2. MS/MS spectra of hydroxylated (top panel) and non-hydroxylated (bottom panel) TRPA1 (322-348).

Conditions: FIH (2 μM FIH, top panel; 0 μM FIH, bottom panel), sodium ascorbate (100 μM), 2-OG (100 μM), ferrous ammonium sulfate (20 μM) in 50 mM Tris buffer (pH 7.5, 37 °C, 1 hour).

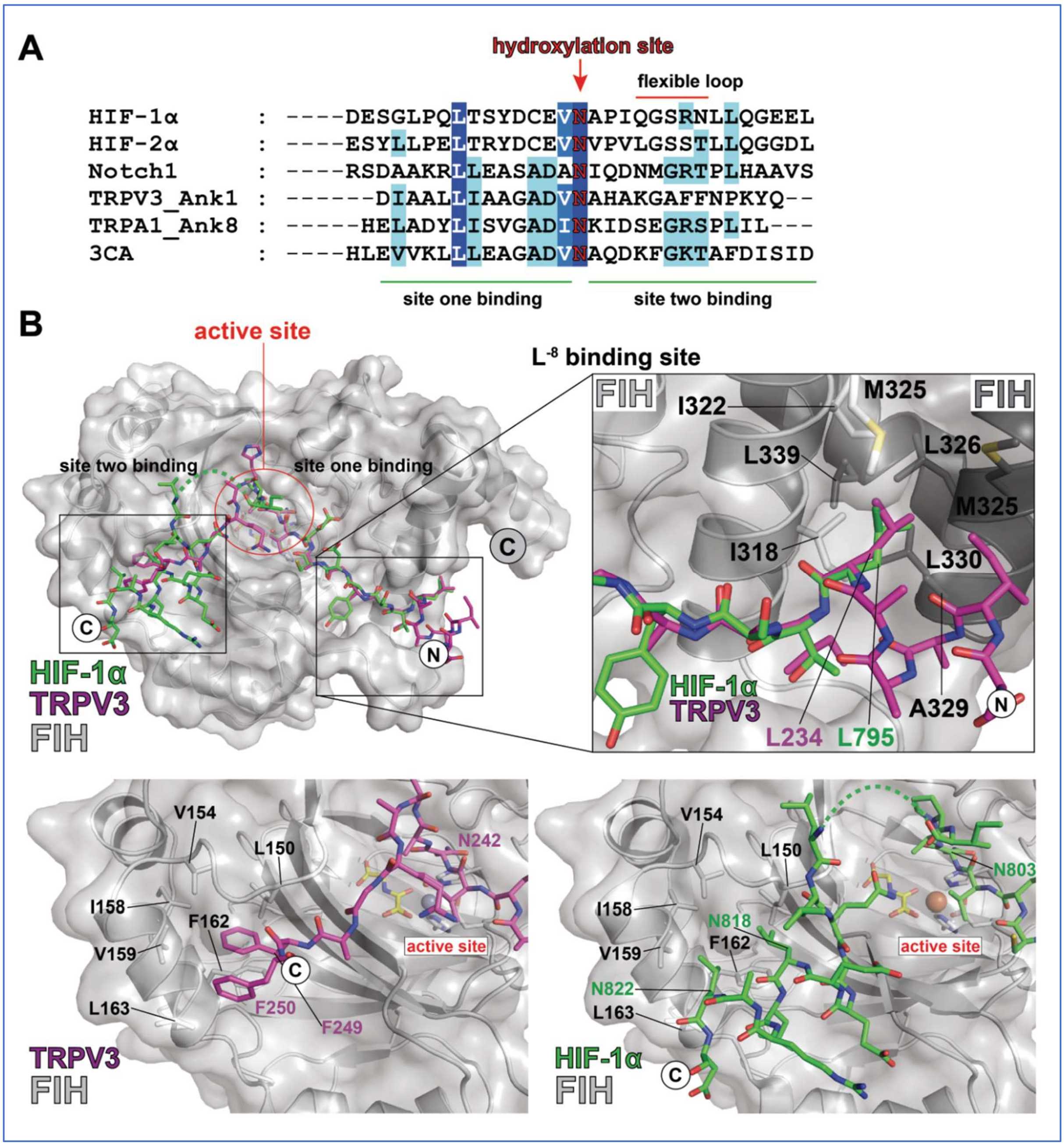

Figure S3. Crystal structure views of FIH in complex with peptides derived from HIF-1α and TRPV3 (229-255) revealing different binding modes of HIF-α and ankyrin substrate fragments to FIH at binding site two. (A) Sequence alignment of selected FIH substrates showing conserved sequences with respect to the regions interacting with FIH substrate binding site one and sequence variations for the regions interacting with binding site two. (B) Views from crystal structures of FIH in complex with TRPV3(229-255) and HIF-1α (PDB code 1H2K) [29] reveal a conserved binding mode to FIH at binding site one and distinctive binding modes to FIH at binding site two.

Supplementary Tables

Table S1. Data collection and refinement statistics for structures of FIH in complex with TRPV3- and TRPA1-derived peptides.

**Datasets**	**FIH**-Zn(II)/TRPV3 Ank1 (220-246) soaked	**FIH**-Zn(II)/TRPV3 Ank1 (229-255), co-crystallised	**FIH**-Zn(II)/TRPA1 Ank8 (313-339), soaked
**PDB code**	**6HA6**	**6H9J**	**6HC8**
**Data Collection**			
Beamline (Wavelength, Å)	DLS I03 (0.9795)	DLS I03 (0.9793)	DLS I03 (0.9795)
Detector	PILATUS 6M-F	PILATUS 6M-F	PILATUS 6M-F
Data Processing	Xia2 3dii (XDS)	Xia2 3d (XDS)	MOSFLM/SCALA
Space group	$P4_12_12$	$P4_12_12$	$P4_12_12$
Cell dimensions			
a,*b*,*c* (Å)	87.1, 87.1, 149.4	86.4, 86.4, 149.6	86.6, 86.6, 148.1
α,β,γ(°)	90, 90, 90	90, 90, 90	90, 90, 90
No. reflections	40834 (2001)*	50768 (2483)*	45517 (6544)*
Resolution (Å)	74.68-1.98 (2.01-1.98)*	38.63-1.83 (1.86-1.83)*	86.86-1.90 (2.00-1.90)*
R_{merge}	0.085 (2.821)*	0.108 (2.928)*	0.100 (2.027)*
I/σI	18.0 (1.4)*	13.7 (1.2)*	13.3 (1.7)*
CC1/2	1.0 (0.6)*	1.0 (0.5)*	0.998 (0.590)*
Completeness (%)	100 (100)*	100 (100)*	100 (100)*

Multiplicity	19.0 (20.2)*	19.4 (20.1)*	17.8 (17.7)*
Wilson B value (Å^2)	49.7	41.7	42.8
**Refinement**	PHENIX	PHENIX	PHENIX
R_{work}/R_{free}†	0.1806/0.2028	0.1658/0.1868	0.1700/0.1903
No. atoms			
-Enzyme	2754	2744	2780
-Substrate	83	158	108
-Ligand	NOG (10)	NOG (10)	NOG (10)
-Water	117	159	147
Avg. B-factors			
Enzyme	65.9	54.1	60.2
Substrate	116.4	55.8	84.8
Ligand	52.0	75.8	45.1
Water	60.3	43.1	57.0
R.m.s deviations			
-Bond lengths (Å)	0.003	0.013	0.007
-Bond angles (°)	0.591	1.065	0.766

* Highest resolution shells in parentheses.

†$R_{factor} = \Sigma_{hkl}||F_{obs}(hkl)|-k|F_{calc}(hkl)||/\Sigma_{hkl}|F_{obs}(hkl)|$, R_{free} is the R_{factor} for ~5%of reflections excluded from the refinement.

Table S2. Buffer and vapour diffusion conditions used for FIH complex crystallisation.

Protein complex	Sample composition	Crystallisation conditions	Vapour diffusion conditions
**FIH** Zn(II)/NOG TRPV3 (220-246)	11 mg/mL FIH (0.27 mM) 50 mM Tris-HCl pH 7.5, peptide (1-2 mM) $Zn(OAc)_2$ (0.5 mM)	0.1 M Hepes pH 7.5, 1.7 M ammonium sulfate, 5.5% PEG400	Sitting drop (300 nL) protein-to-well ratio 2:1, 293K
**FIH** Zn(II)/NOG TRPA1 (313-339)	11 mg/mL FIH (0.27 mM) in 50 mM Tris-HCl pH 7.5, peptide (1-2 mM) $Zn(OAc)_2$ (0.5 mM)	0.1 M Hepes pH 7.5, 1.6 M ammonium sulfate, 6% PEG400	Sitting drop (300 nL) protein-to-well ratio 2:1, 293K
**FIH** Zn(II)/NOG TRPV3 (229-255)	11 mg/mL FIH (0.27 mM) in 50 mM Tris-HCl pH 7.5, peptide (1-2 mM) $Zn(OAc)_2$ (0.5 mM)	0.1 M Hepes pH 7.5, 1.8 M ammonium sulfate, 4.5% PEG400	Sitting drop (300 nL) protein-to-well ratio 1:2, 293K

Peptides were prepared with C-terminal amides.
